# International collaboration for global accessibility of COVID-19 vaccines

**DOI:** 10.1093/nsr/nwaa147

**Published:** 2020-07-08

**Authors:** Qi Zhou

**Affiliations:** Institute for Stem Cell and Regeneration, Chinese Academy of Sciences, China Editorial Board Member of NSR

The novel coronavirus has been spreading and has currently affected over 200 countries and territories across the world. However, up to now, there is still no cure for COVID-19. Not surprisingly, the vaccine is considered the ultimate solution to the coronavirus pandemic and it is believed that there has never been a more urgent task than creating broad immunity against the coronavirus.

Currently, intense global research and development (R&D) activities have been conducted to develop vaccines against coronavirus. China has made outstanding contributions in this effort. For example, a new study published on 22 May 2020 in the journal *The Lancet* revealed that nearly all of 108 healthy adults aged 18 to 60, who did not have COVID-19, were vaccinated in China and found to develop antibodies that bound to SARS-CoV-2. Approximately half of the participants in the low- and middle-dose groups and three-quarters of participants in the high-dose group developed ‘neutralizing antibodies’, which bind to and disable the virus to prevent it from infecting cells. Besides this clinical trial in China, four other vaccines have also entered phase two clinical trials. China is now clearly in the forefront of global vaccine R&D. The United States and European Union are also promoting COVID-19 vaccine research and development, with potential vaccines in the pipeline for clinical trials. However, it is worrisome that several countries are battling against one another to be the first to find a cure or a vaccine, bringing a nationalist element to the worldwide crisis.

Taking into account that the failure rate of vaccine research and development is considerably high, and that failure may occur at any stage of vaccine development, stronger and broader international coordination and cooperation among COVID-19 vaccine developers, investors, policymakers and governments should be promoted, so as to ensure successful development of a vaccine as soon as possible. In fact, the pandemic is the common enemy of all mankind and affects all countries and individuals. No country can protect itself without cooperating with others. Only when all countries unite can the COVID-19 pandemic be addressed ultimately.

With that in mind, the global vaccine community has collectively mobilized technical and financial support, and its members have started cooperating with each other. For example, on 3 June 2020, the Dutch Ministry of Health announced that four of Europe's largest economies have forged an alliance to accelerate the development and production of a vaccine ‘on European soil’ against the coronavirus. China and Canada have been actively collaborating in mounting responses against the COVID-19 pandemic. In May, a Chinese vaccine company announced its collaboration with the Canadian National Research Council on clinical trials for the Recombinant Novel Coronavirus Vaccine (Ad5-nCoV). Nevertheless, countries around the world should still choose cooperation over confrontation, in order to truly develop effective drugs and vaccines against COVID-19 as early as possible.

When promising late-stage vaccine candidates are about to be used, it is extremely important to consider the availability and accessibility of vaccines for all affected areas. Taking into account that the coronavirus is extremely transmissible or lethal, governments need to guarantee both accessibility and affordability of vaccines for everyone, in particular for the poor and people living in areas with low resources.

From the global perspective, it is necessary to make vaccines accessible to developing and poor countries in the world. To be specific, developed countries, which have robust vaccine R&D and manufacturing capacities, could consider setting aside a portion of each production cycle of COVID-19 vaccines for stockpiling and/or use, as appropriate, by developing and poor countries. Governments around the world should continue to work with one another with the purpose of ensuring that an adequate supply of COVID-19 vaccines are made available to developing and poor countries at the same time as developed countries, in order to safeguard global public health.

China is an advocate and practitioner of building a community with a shared future for mankind. On the basis of this philosophy, Xi Jinping, the President of China, has announced that COVID-19 vaccines developed by China will be used as a global public product. This implies that China is willing to cooperate with other countries in the world to jointly develop vaccines and contribute its research findings to the world. Thus, if more researchers, developers and countries follow China's example and make vaccines available for more people across the world, it will strengthen our capacity as a species to tackle the COVID-19 pandemic and future pandemics.

